# Frequency of unnecessary prenatal diagnosis of hemoglobinopathies: A large retrospective analysis and implication to improvement of the control program

**DOI:** 10.1371/journal.pone.0283051

**Published:** 2023-04-14

**Authors:** Kritsada Singha, Supawadee Yamsri, Attawut Chaibunruang, Hataichanok Srivorakun, Kanokwan Sanchaisuriya, Goonnapa Fucharoen, Supan Fucharoen

**Affiliations:** 1 Centre for Research and Development of Medical Diagnostic Laboratories, Faculty of Associated Medical Sciences, Khon Kaen University, Muang, Khon Kaen, Thailand; 2 Faculty of Medicine, Mahasarakham University, Mahasarakham, Thailand; University of Naples Federico II, ITALY

## Abstract

**Objective:**

To determine the frequency and etiology of unnecessary prenatal diagnosis for hemoglobinopathies during 12 years of services at a single university center in Thailand.

**Methods:**

We conducted a retrospective cohort analysis of prenatal diagnosis during 2009–2021. A total of 4,932 couples at risk and 4,946 fetal specimens, including fetal blood (5.6%), amniotic fluid (92.3%), and chorionic villus samples (2.2%) were analyzed. Identification of mutations causing hemoglobinopathies was carried out by PCR-based methods. Maternal contamination was monitored by analysis of the D1S80 VNTR locus.

**Results:**

Among 4,946 fetal specimens, 12 were excluded because of poor PCR amplification, maternal contamination, non-paternity, and inconsistency of the results of the fetuses and parents. Breakdown of 4,934 fetuses revealed 3,880 (78.6%) at risk for the three severe thalassemia diseases, including β-thalassemia major, Hb E-β-thalassemia, and homozygous α^0^-thalassemia, 58 (1.2%) at risk for other α-thalassemia diseases, 168 (3.4%) at risk for β^+^-thalassemia, 109 (2.2%) at risk for high Hb F determinants, 16 (0.3%) at risk for abnormal Hbs, and 294 (6.0%) with no risk of having severe hemoglobinopathies. The parents of 409 (8.3%) fetuses had inadequate data for fetal risk assessment. Overall, we encountered unnecessary prenatal diagnostic requests for 645 (13.1%) fetuses.

**Conclusions:**

The frequency of unnecessary prenatal diagnosis was high. This could lead to unnecessary risk of complications associated with fetal specimen collection, psychological impacts to the pregnant women and their families, as well as laboratory expenses and workload.

## Introduction

Hemoglobinopathies are the most common inherited hemoglobin (Hb) disorders. Approximately 7% of the world population are carriers, and 300,000–400,000 babies with severe forms of hemoglobinopathies are born each year [1, 2]. In Thailand, the prevalence of 20–30% α-thalassemia, 3–9% β-thalassemia, 20–30% Hb E, and 1–8% Hb Constant Spring have been reported [3]. Other forms of hemoglobinopathies are occasionally documented [4, 5]. The high prevalence of these genetic abnormalities can lead to diverse heterogeneity of thalassemia and hemoglobinopathies and several complex thalassemia syndromes. It has been estimated that about 0.6% of the Thai population are suffered from thalassemia diseases, and about 10,000 affected births are born each year [3].

**Fig 1:**
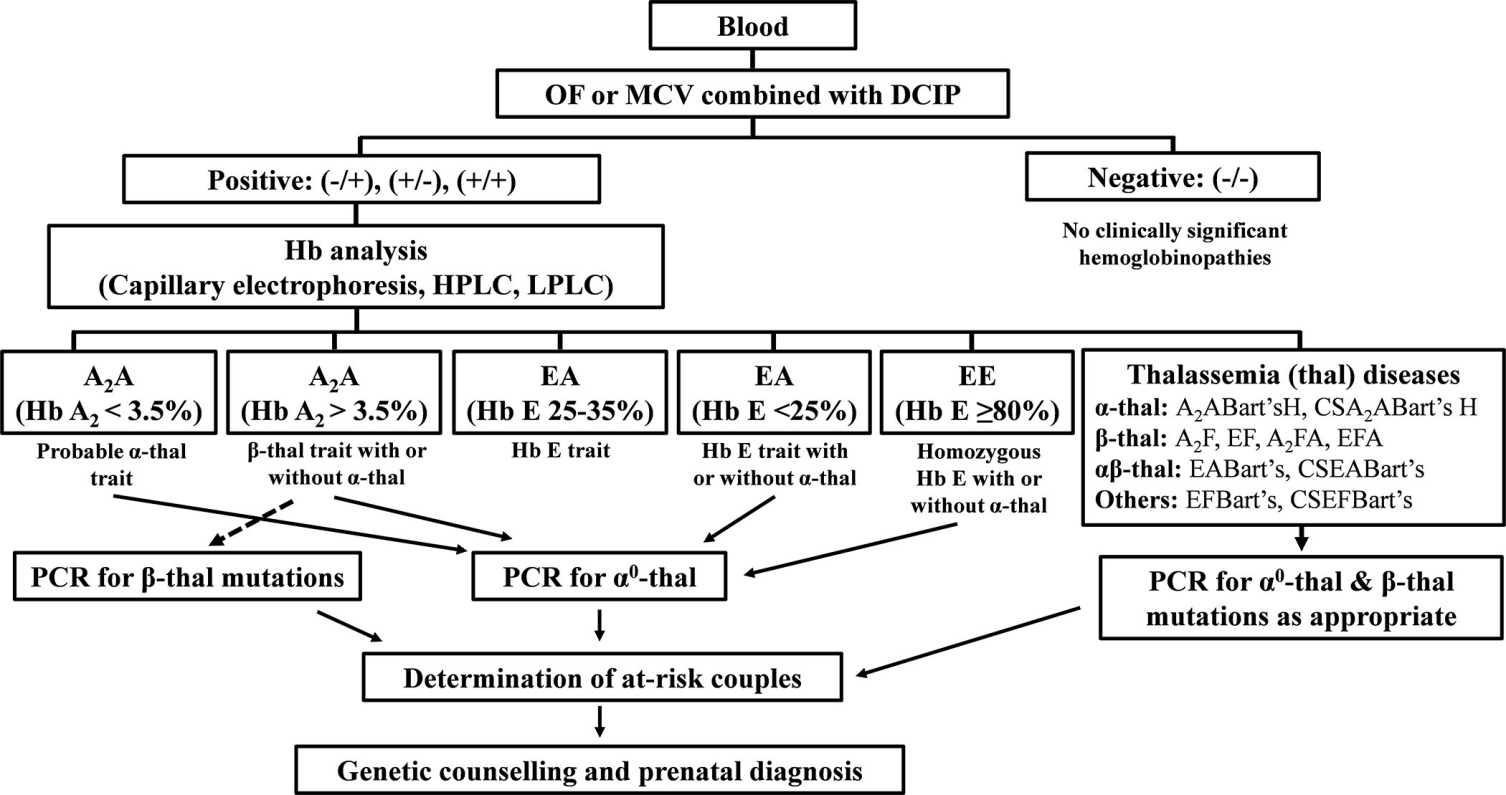
Screening diagram for carriers of α^0^-thalassemia, β-thalassemia and Hb E in Thailand using the combined osmotic fragility (OF) test, MCV and dichlorophenolindophenol (DCIP) test. (Modified from Fucharoen G et al 2004 [6] and Sanchaisuriya K et al 2005 [7]).

In Thailand, a program has been established for the prevention and control of the three severe thalassemia diseases, Hb Bart’s hydrops fetalis (homozygous α^0^-thalassemia), homozygous β-thalassemia, and Hb E-β-thalassemia. Carrier screening, genetic counseling, and prenatal diagnosis (PND) are carried out to prevent the births of new cases with these severe diseases [3]. As shown in **Fig 1**, initial screening for target carriers of α^0^-thalassemia, β-thalassemia, and Hb E is usually performed using a combined mean corpuscular volume (MCV) with a cut-off value of 80 fL, mean corpuscular hemoglobin (MCH) with a cut-off value of 27 pg, or osmotic fragility (OF) test and a dichlorophenolindophenol (DCIP) test for Hb E. While those with a negative screen could be excluded, the positive-screened couples must be further investigated by Hb and DNA analyses. Genetic counseling and prenatal diagnosis are then offered to those couples at risk of having fetuses with severe thalassemia diseases [6, 7].

Prenatal screening of thalassemia can effectively reduce the incidence of severe thalassemia in the population [8, 9]. The overall performance of the prevention and control program conducted at our center at Khon Kaen University in northeast Thailand during 1993–2008 has been reported [9]. However, unnecessary prenatal diagnosis has been continuously noted in routine services. In this study, we report a retrospective cohort study on the frequency and etiology of the unnecessary prenatal diagnosis of hemoglobinopathies for 12 years of services during 2009–2021.

## Materials and methods

### Subjects, hematological, and DNA analyses

This retrospective study was conducted ethically in accordance with the Declaration of Helsinki and ethical approval of the study protocol was obtained from the Institutional Review Board (IRB) of Khon Kaen University, Khon Kaen, Thailand. The study has been granted an exemption from requiring written informed consent of the participants by the IRB of Khon Kaen University, Thailand (HE622173). Retrospective data of 4,932 couples and 4,946 fetal tissues (with 14 twin pregnancies) encountered for prenatal diagnosis of hemoglobinopathies from January 2009 to December 2021 were recruited. The fetal tissues collected by experienced obstetricians included chorionic villus sampling (CVS), amniotic fluid, and fetal blood specimens. Fetal and parent DNA were prepared using a GF-1 Blood DNA extraction kit (Vivantis, Malaysia). We routinely monitor maternal contamination of fetal DNA specimens by analyzing a variable number of tandem repeats (VNTR) polymorphism of the D1S80 locus [9]. Hb F cell staining was performed for all fetal blood specimens [10]. Hematological parameters were collected on a standard blood cell counter. Hb analysis was performed using an automated high-performance liquid chromatography (HPLC) analyzer (Variant^TM^, Bio-Rad laboratories, Hercules, CA, USA) or the capillary electrophoresis (CapillaryS 2; Sebia, Lisses, France). Common α-thalassemia (—^SEA^,—^THAI^, -α^3.7^, -α^4.2^, Hb Constant Spring (HBA2:c.427T>C), Hb Paksé (HBA2:c.429A>T), β-thalassemia, Hb E, abnormal Hbs and high Hb F determinants mutations found in Thailand were routinely identified by PCR-based methods as described elsewhere [4, 5, 9, 11, 12].

### Determination of couples at risk

Based on the results of thalassemia screening of the parents, pregnancies at risk of having fetuses with thalassemia diseases are determined as shown in **Table 1** [6, 7, 9, 13, 14]. For those of couples with the risks of having fetuses with severe thalassemia diseases, genetic counseling and PND are offered. For those with the risks of having fetuses with non-severe thalassemia diseases, genetic counseling is provided but PND is not necessary.

**Table 1:** Parental carrier state combinations that give rise to the fetal risk of having severe thalassemia (serious risk) requiring PND, less serious risk and minimal risk with no need for PND encountered in this study.

	Mother											
**Father**	**Carrier of**	**β^0^-thalassemia**	**β^+^-thalassemia**	**Hb E**	**δβ^0^-thalassemia**	**Hb Lepore**	**HPFH**	**Other abnormal Hbs** *	**α^0^-thalassemia**	**α^+^-thalassemia**	**Hb Constant Spring**	**Not a carrier**
	**β^0^-thalassemia**											
	**β^+^-thalassemia**											
	**Hb E**											
	**δβ^0^-thalassemia**											
	**Hb Lepore**											
	**HPFH**											
	**Other abnormal Hbs** *											
	**α^0^-thalassemia**											
	**α^+^-thalassemia**											
	**Hb Constant Spring**											
	**Not a carrier**											

* Including Hb Tak, Hb C, Hb Hope, Hb Pyrgos, Hb J-Bangkok, and Hb Korle-Bu

**Black: Serious risk**: Need genetic counselling and PND is offered.

**Grey: Less serious risk**: Need genetic counselling, further investigation may be required but PND is unnecessary.

**White: Minimal risk**.

## Results

For 12 years between January 2009-December 2021, we obtained consecutively 4,946 fetal specimens for hemoglobinopathies investigation. Of these 4,946 specimens, 12 were excluded from the study, including five specimens with PCR failure due to too small amounts of fetal specimens, four samples with maternal contamination as monitored by the D1S80 VNTR analysis, two samples with suspected non-paternity based on VNTR analysis, and 1 sample with inconsistency results of the fetus and the parents. The remaining 4,934 fetal specimens including CVS (n = 107, 2.2%), amniotic fluid (n = 4,554, 92.3%) and fetal blood (n = 273, 5.5%) were further analyzed. As shown in **Table 2**, among these 4,934 PND requests, there were 3,706 (75.1%) requests with previously known parental mutations prior to fetal sampling, and 1,228 (24.9%) requests with unknown parental mutations. We found that 3,542 of the former 3,706 (95.6%) fetuses were at risk of having the three targeted severe thalassemia diseases, i.e., homozygous α^0^-thalassemia (Hb Bart’s hydrops fetalis), β-thalassemia major and Hb E-β^0^-thalassemia. In contrast, only 338 of the latter 1,228 (27.5%) fetuses were at risk for these three severe thalassemia diseases.

**Table 2:** Numbers of PND requests for hemoglobinopathies during 2009–2021, grouped according to necessary (n = 3,880) and unnecessary (n = 645) requests and requests with inadequate data for fetal risk assessment (n = 409). Numbers of requests with known and unknown parental mutations prior to fetal sampling and the risks for hemoglobinopathies of the fetuses are listed. CS = Hemoglobin Constant Spring, thal = thalassemia.

PND groups and at-risk diseases	Total number (%)	Parental mutations prior to fetal sampling	
		Known (%)	Unknown (%)
**Necessary prenatal diagnosis**	**3,880 (78.6)**	**3,542 (95.6)**	**338 (27.5)**
Severe thal diseases (homozygous α^0^-thal, β-thal major and Hb E-β^0^-thal)			
**Unnecessary prenatal diagnosis**	**645 (13.1)**	**164 (4.4)**	**481 (39.2)**
Other α-thal diseases (Hb H, Hb H-CS & homozygous Hb CS)	58 (1.2)	26 (0.7)	32 (2.6)
β^+^-thal	168 (3.4)	50 (1.3)	118 (9.6)
High Hb F determinants	109 (2.2)	65 (1.8)	44 (3.6)
Abnormal hemoglobins	16 (0.3)	7 (0.2)	9 (0.7)
No risk for thal & hemoglobinopathies	294 (6.0)	16 (0.4)	278 (22.6)
**Inadequate data for fetal risk assessment**	**409 (8.3)**	**0**	**409 (33.3)**
**Total**	**4,934 (100)**	**3,706 (75.1)**	**1,228 (24.9)**

Altogether, among the 4,934 fetuses, 3,880 (78.6%) were found to be at risk for the three severe thalassemia diseases targeted by the national prevention and control program. A total of 645 (13.1%) fetuses were classified in this study as a group of unnecessary PND as they were at risk of having other mild forms of thalassemia, including α-thalassemia diseases (Hb H disease, Hb H-Constant Spring disease, and homozygous Hb Constant Spring) (n = 58, 1.2%), β^+^-thalassemia (n = 168, 3.4%), high Hb F determinants (n = 109, 2.2%), and abnormal Hbs (n = 16, 0.3%). Of these 645 fetuses, 294 (6.0%) were found to have no risk for thalassemia and hemoglobinopathies. Unfortunately, available data on the parents were inadequate in the remaining 409 (8.3%) fetuses for genetic risk assessments. However, we still performed DNA analyses for all of them to determine the fetal thalassemia genotypes. The molecular defects causing α-thalassemia, β-thalassemia, high Hb F determinants, and abnormal Hbs identified were summarized in **S1–S4 Tables**, respectively.

**Table 3** summarizes the fetal genetic risks of thalassemia diseases based on genotypes of the parents and the outcome of PND for all 4,934 fetuses. A total of 3,880 (78.6%) fetuses were at risk for severe thalassemia diseases, including Hb Bart’s hydrops fetalis caused by homozygous α^0^-thalassemia, Hb E-β^0^-thalassemia, homozygous β^0^-thalassemia, compound heterozygous for β^0^/β^+^-thalassemia and compound δβ^0^/β^0^-thalassemia. These are targeted diseases of the national prevention and control program for thalassemia in Thailand. As shown in the table, the two most common ones are Hb E-β-thalassemia disease and Hb Bart’s hydrops fetalis. Others forms of thalassemia are less common. PND identified 1,074 (27.7%) affected fetuses, 1,987 (51.2%) unaffected carriers and 819 (21.1%) normal fetuses, quite agreed with the 1:2:1 theoretical ratio of a genetic recessive disorder.

**Table 3:** Proportions of the 4,934 fetuses and at-risk diseases in each PND group and the PND outcomes. CS = Hemoglobin Constant Spring, thal = thalassemia.

PND groups and diseases at risk of the fetuses	Total (%)		PND outcomes	
		Affected (%)	Unaffected carrier (%)	Normal (%)
***Necessary prenatal diagnosis***				
Hb E-β^0^-thal with couples of β^0^-thal carrier and Hb E carrier	1,961	520 (26.5)	1,007 (51.4)	434 (22.1)
Hb E-β^0^-thal with couples of β^0^-thal carrier and homozygous Hb E	254	108 (42.5)	146 (57.5)	0
Hb Bart’s hydrops fetalis (homozygous α^0^-thal)	1,503	401 (26.7)	749 (49.8)	353 (23.5)
Homozygous β^0^-thal	90	26 (28.9)	46 (51.1)	18 (20.0)
β^0^-thal/β^+^-thal	41	6 (14.6)	23 (56.1)	12 (29.3)
Hb Bart’s hydrops fetalis & Hb E-β^0^-thal	17	10 (58.8)	6 (35.3)	1 (5.9)
Homozygous β^0^-thal & Hb E-β^0^-thal	8	3 (37.5)	5 (62.5)	0
δβ^0^-thal/β^0^-thal	6	0	5 (83.3%)	1 (16.7%)
**Total**	**3,880 (78.6)**	**1,074 (27.7)**	**1,987 (51.2)**	**819 (21.1)**
***Unnecessary prenatal diagnosis***				
**Other α-thal diseases**	**58**	**38 (65.5)**	**16 (27.6)**	**4 (6.9)**
Homozygous Hb Constant Spring	26	20 (76.9)	5 (19.2)	1 (3.8)
Hb H disease	17	10 (58.8)	5 (29.4)	2 (11.8)
Hb H-Constant Spring disease	15	8 (53.3)	6 (40.0)	1 (6.7)
**β^+^-thal diseases**	**168**	**38 (22.6)**	**95 (56.5)**	**35 (20.8)**
Hb E-β^+^-thal	159	34 (21.4)	91 (57.2)	34 (21.4)
Homozygous β^+^-thal	7	3 (42.9)	3 (42.9)	1 (14.3)
Hb E-β^+^-thal & Homozygous β^+^-thal	2	1 (50.0)	1 (50.0)	0
**High Hb F determinants**	**109**	**34 (31.2)**	**57 (52.3)**	**18 (16.5)**
Hb E-δβ^0^-thal	82	22 (26.8)	48 (58.5)	12 (14.6)
Hb E-HPFH	12	5 (41.7)	4 (33.3)	3 (25.0)
Homozygous δβ^0^-thal	4	2 (50.0)	1 (25.0)	1 (25.0)
δβ^0^-thal/HPFH	3	2 (66.7)	1 (33.3)	0
δβ^0^-thal/β^+^-thal	3	1 (33.3)	2 (66.7)	0
HPFH /β^+^-thal	3	0	1 (33.3)	2 (66.7)
HPFH/β^0^-thal	2	2 (100)	0	0
**Abnormal hemoglobins**	**16**	**4 (25.0)**	**9 (56.3)**	**3 (18.8)**
Hb Tak/Hb E or Hb Tak/β^0^-thal	4	2 (50.0)	2 (50.0)	0
Hb Lepore/Hb E or Hb Lepore carrier	4	0	3 (75.0)	1 (25.0)
Hb Hope/Hb E or Hb Hope/β^0^-thal	2	1 (50.0)	0	1 (50.0)
Hb Pyrgos/Hb E	2	1 (50.0)	1 (50.0)	0
Hb J Bangkok/Hb E	2	0	1 (50.0)	1 (50.0)
Hb C/Hb E	1	0	1 (100)	0
Hb Korle-Bu/Hb E	1	0	1 (100)	0
**No risk for thal & hemoglobinopathies**	**294**	**0**	**83 (28.2)**	**211 (71.8)**
Negative for α^0^-thal in one or both parents	207	0	16 (7.7)	191 (92.3)
Non-β-thal carrier	50	0	45 (90.0)	5 (10.0)
Falsely high Hb A_2_ level	30	0	18 (60.0)	12 (40.0)
Invalid result	7	0	4 (57.1)	3 (42.9)
**Total**	**645 (13.1)**	**114 (17.7)**	**260 (40.3)**	**271 (42.0)**
***Data inadequate for fetal risk assessment***				
Requests for α^0^-thal	242	34 (14.0)	76 (31.4)	132 (54.5)
Requests for β-thal and Hb E	133	19 (14.3)	51 (38.3)	63 (47.4)
Requests with uncharacterized β-thal mutations	24	0	11 (45.8)	13 (54.2)
Requests for α^0^-thal, β-thal and Hb E	10	0	7 (70.0)	3 (30.0)
**Total**	**409 (8.3)**	**53 (13.0)**	**145 (35.5)**	**211 (51.6)**

**Fig 2:**
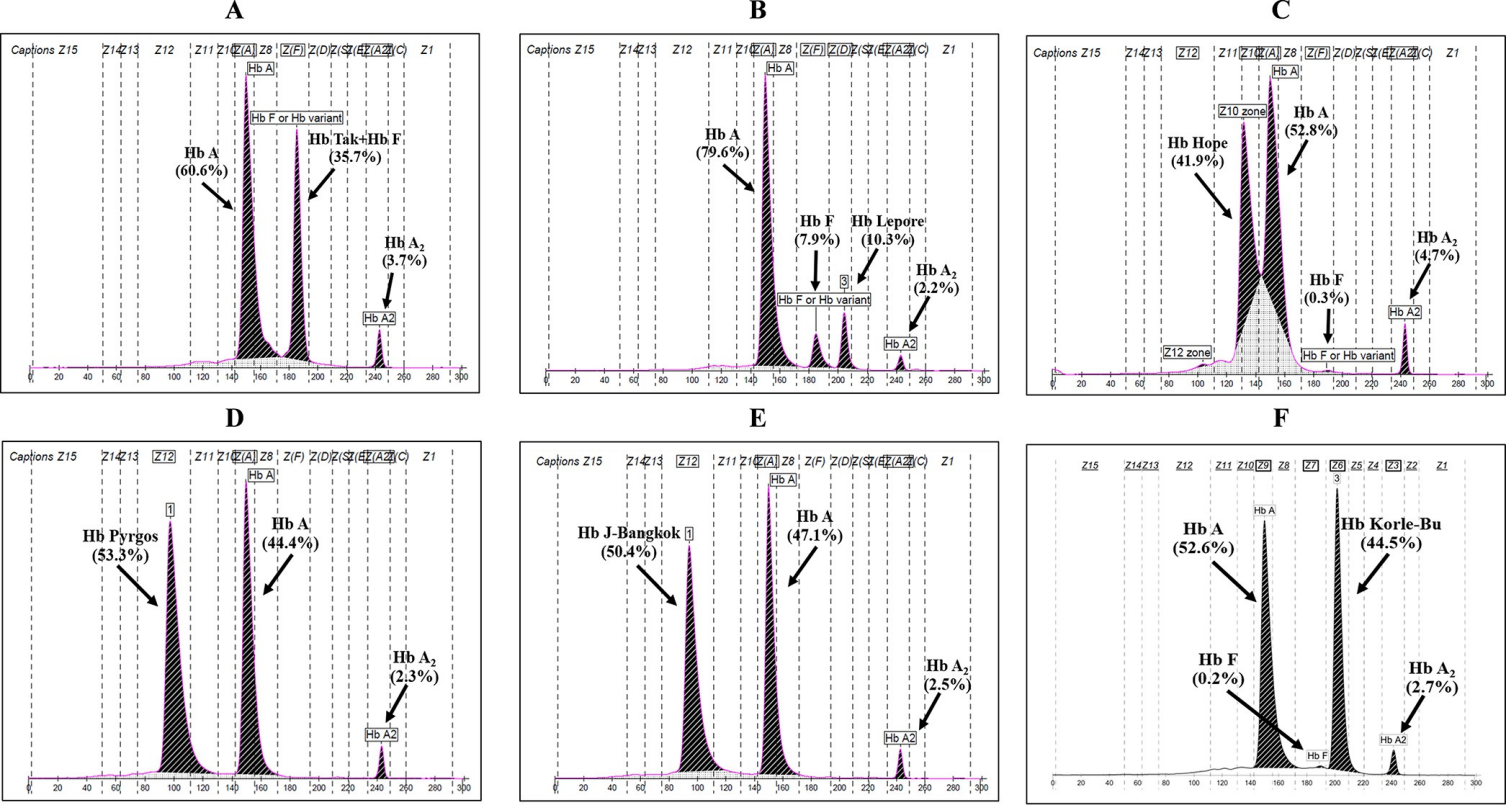
Representative Hb analysis by capillary electrophoresis of various abnormal Hbs encountered in the unnecessary PND group, including carriers of Hb Tak (**A**), Hb Lepore (**B**), Hb Hope (**C**), Hb Pyrgos (**D**), Hb J-Bangkok (**E**), and Hb Korle Bu (**F**). Normal and abnormal Hbs are indicated.

We considered several mild forms of thalassemia diseases as unnecessary PND since they are not the targets of a prevention and control program. They have been identified in 645 (13.1%) fetuses. These included 58 (1.2%) fetuses at risk of having other α-thalassemia diseases, namely homozygous Hb Constant Spring (n = 26), Hb H disease (n = 17), and Hb H-Constant Spring (n = 15), 168 (3.4%) fetuses for β^+^-thalassemia disease, 109 (2.2%) fetuses for high Hb F determinants including δβ^0^-thalassemia and hereditary persistence of fetal Hb (HPFH), and 16 (0.3%) fetuses for several abnormal Hbs in association with Hb E or β-thalassemia including Hb Tak (HBB:c.440_441dupAC) (n = 4), Hb Lepore (δβ-hybrid Hb)(n = 4), Hb Hope (HBB:c.410G>A) (n = 2), Hb Pyrgos (HBB:c.251G>A) (n = 2), Hb J-Bangkok (HBB:c.170G>A) (n = 2), Hb C (HBB:c.19G>A) (n = 1) and Hb Korle-Bu (HBB:c.220G>A) (n = 1), all of which are clinically innocuous and have been reported before in Thai population [4]. **Fig 2** demonstrated representatively Hb analysis of abnormal Hbs encountered in this unnecessary PND group. In addition, 294 (6.0%) fetuses were found to have no risk for thalassemia disease. Unlike those with the risks for severe thalassemia diseases mentioned above, on this group of unnecessary PND, fetal tissue analysis identified affected fetuses (n = 114, 17.7%), unaffected carriers (n = 260, 40.3%), and normal fetuses (n = 271, 42.0%). Similar proportions of PND outcomes were noted on the 409 (8.3%) fetuses who were requested for PND with inadequate information for fetal risk assessments, i.e., 55 (13.4%) affected fetuses, 155 (37.9%) unaffected carriers, and 199 (48.7%) normal fetuses.

## Discussion

The serious complication in sampling of a fetal specimen is miscarriage. The overall loss rate of chorionic villus sampling is approximately 2%, with an adjusted procedure-related loss rate of approximately 1:400. The procedure-related loss rate of amniocentesis and cordocentesis is approximately 1:300 to 1:500 and 1.4%, respectively [15]. Other possible complications include infection, amnionic fluid leakage, chorioamnionitis, cord vessel bleeding, fetal-maternal bleeding, and fetal bradycardia. Moreover, psychological impacts, especially anxiety in pregnant women, are usually associated with invasive prenatal diagnosis [15–18]. It is therefore recommended to carry out invasive PND only in essential cases to minimize these adverse effects. Therefore, for hemoglobinopathies which are highly heterogeneous, it is essential to define the targeted diseases in PND. In Thailand, three severe thalassemia diseases, namely Hb Bart’s hydrops fetalis (homozygous α^0^-thalassemia), homozygous β-thalassemia, and Hb E-β-thalassemia have been set as the targets for the prevention and control program [3, 9]. Couples at risk of having fetuses with these three thalassemia diseases are offered PND, genetic counseling and termination of pregnancy with affected fetuses. Other thalassemia syndromes are not included in the program.

We have demonstrated that this approach has been effective in the prevention of new cases of severe thalassemia in northeast Thailand [9]. However, we have yet encountered unnecessary PND during 2009–2021 services at our referral center in northeast Thailand. This indicates that unnecessary PND is not an uncommon event and needs to be addressed in a routine setting. As shown in **Table 2**, retrospective analysis of 4,934 fetuses at PND for hemoglobinopathies, we identified that 3,880 (78.6%) fetuses were at risk of having the three targeted severe thalassemia diseases; homozygous α^0^-thalassemia, β-thalassemia major and Hb E-β^0^-thalassemia. Of interest are the findings of PND requests of 645 (13.1%) fetuses for non-severe forms of hemoglobinopathies and 409 (8.3%) PND requests with inadequate data for fetal risk assessment. It is noteworthy that when these 4,934 PND requests were grouped according to the knowledge of the parental mutations prior to fetal sampling, we found that majority of PND requests without prior knowledge of the parental mutations were not related to the targeted thalassemia diseases, i.e., only 27.5% of the fetuses were at risk of having the three targeted severe thalassemia diseases. It is therefore recommended to have a PND request only after the parental mutations have been identified.

As shown in **Table 3**, it is not unexpected that for the true risk group of 3,880 fetuses, we obtained the average PND outcome of affected fetuses, unaffected carriers, and normal fetuses in 27.7%, 51.2%, and 21.1%, respectively, quite corresponding to the expected ratio of 1:2:1 for a recessive genetic disorder. The relatively small higher proportions of affected fetuses (27.7% *versus* 25.0% theoretical value) and unaffected carriers (51.2% *versus* 50.0% theoretical value) and lower proportion of normal fetuses (21.1% *versus* 25.0% theoretical value) could be explained. For some genotypes, e.g., couples at risk who were β-thalassemia trait and homozygous Hb E (Table 3, n = 254) would have a 50% chance of having affected fetuses and unaffected fetal carriers but have no chance of having normal fetuses. In addition, many couples at risk carried more than one thalassemia genotypes and therefore had the risk of having fetuses with more than one severe thalassemia diseases, e.g., both Hb Bart’s hydrops fetalis & Hb E-β^0^-thalassemia and homozygous β^0^-thalassemia & Hb E-β^0^-thalassemia.

In the unnecessary PND group, we have noted that 58 fetuses were at risk of having Hb H, Hb H-Constant Spring, or homozygous Hb Constant Spring. All these diseases are usually associated with mild thalassemia intermedia phenotype rather than severe thalassemia syndrome and are not the targets of prevention and control programs [1, 3, 9]. However, it has been reported that fetuses with homozygous Hb Constant Spring may be suffered from severe fetal anemia, cardiomegaly, and hydrops fetalis, which can be effectively treated with intrauterine transfusion [19–21]. Therefore, although not targeted in the national prevention and control program, PND of homozygous Hb Constant Spring might be taken into consideration. This is not for termination of pregnancy but for allowing earlier and appropriate management of such cases. This PND of homozygous Hb Constant Spring can be done easily using fetal Hb or DNA analyses [22, 23].

In a group of β^+^-thalassemia disease (n = 168), we recommend screening of β-thalassemia mutation in the couple at risk before fetal sampling. It has been documented that approximately 25% of β-thalassemia in Thailand is β^+^-thalassemia [24, 25]. Although compound heterozygous for β^0^-/β^+^-thalassemia is associated with the severe clinical symptom of transfusion-dependent thalassemia, homozygous β^+^-thalassemia and Hb E-β^+^-thalassemia are associated with mild β-thalassemia phenotype [1, 26, 27]. This is also the case for those with high Hb F determinants (n = 109), including δβ^0^-thalassemia and HPFH disorders. Only compound heterozygous for δβ^0^-thalassemia/β^0^-thalassemia is associated with severe β-thalassemia syndrome, requiring PND during pregnancy [28]. Other forms of high Hb F determinants including Hb E-δβ^0^-thalassemia, Hb E-HPFH, homozygous δβ^0^-thalassemia, δβ^0^-thalassemia/HPFH, and HPFH/β-thalassemia are all associated with mild phenotype of thalassemia or clinically normal [5, 29–32]. As for β^+^-thalassemia, screening of these high Hb F determinants in the couple at risk before making a decision on PND is recommended. The high Hb F characteristic of these genetic defects may be confused with the diagnosis of β-thalassemia. Fortunately, unlike β-thalassemia with elevated Hb A_2_, carriers of these high Hb F determinants are usually associated with normal Hb A_2_ and elevated Hb F levels, and molecular screening of these cases can be done using multiplex PCR assays [5, 33].

Of the 16 fetuses at risk of having abnormal Hbs, 14 were at risk for compound heterozygosity for abnormal Hb/Hb E and 2 were at risk for abnormal Hb/β^0^-thalassemia. Seven different abnormal Hbs were identified, including Hb Tak, Hb Lepore (δβ hybrid Hb), Hb Pyrgos, Hb J-Bangkok, Hb C, Hb Korle-Bu, and Hb Hope. They were at risk for Hb E-Hb Lepore or heterozygous Hb Lepore (n = 4), Hb E-Hb Tak (n = 3), Hb E-Hb Pyrgos (n = 2), Hb E-Hb J-Bangkok (n = 2), Hb E-Hb C (n = 1), Hb E-Hb Korle-Bu (n = 1), Hb E-Hb Hope (n = 1), Hb Hope-β^0^-thalassemia (n = 1), and Hb Tak-β^0^-thalassemia (n = 1). In fact, most of these Hb variants are clinically innocuous, even found in combination with Hb E or β-thalassemia [4, 26, 34–38]. Association of Hb Tak with δβ^0^-thalassemia may alternatively lead to secondary erythrocytosis rather than severe thalassemia syndrome due to the high oxygen affinity characteristics of Hb Tak and Hb F [39]. Therefore, PND of these genetic combinations may be unnecessary. Screening and diagnosis of these Hb variants in the couples at risk prior to PND is recommended. As shown in **Fig 2**, these Hb variants can be easily recognized by routine Hb analysis using capillary electrophoresis or combined capillary electrophoresis and Hb-HPLC analysis before being further confirmed by DNA analysis [4]. However, care should be taken into consideration in the interpretation of Hb analysis results. For example, the carrier of Hb Tak may have borderline Hb A_2_ and Hb Tak co-migrates with F, possibly leading to misinterpretation (**Fig 2A**). Likewise, elevated Hb F with Hb Lepore (**Fig 2B**) and a falsely increased Hb A_2_ in Hb Hope (**Fig 2C**) might also lead to a misdiagnosis of β-thalassemia carrier.

Unexpectedly, we have encountered 294 (6.0%) PND requests for fetuses with no risk of thalassemia disease. Hb and molecular analysis identified four events. The most common one is the PND requests for Hb Bart’s hydrops fetalis without α^0^-thalassemia in one or both parents (n = 207). Requests for β-thalassemia disease were found in 50 couples with non-β-thalassemia carrier (including 34 homozygous Hb E, 8 with Hb A_2_ < 3.5%, and 8 Hb E carriers). An elevated Hb A_2_ in Hb E disorders can be confused with the diagnosis of β-thalassemia carrier in routine thalassemia diagnostic [40]. Likewise, cases of homozygous Hb E with elevated Hb F may also lead to a misdiagnosis of Hb E-β^0^-thalassemia disease, requiring further molecular differentiation. Alternatively, we have demonstrated that the EE score, an arbitrary formula based on Hb A_2_ and Hb F expression, can help in the differentiation of these two common conditions [41]. The remaining 7 PND requests had invalid results due to pre-analytical, analytical, and post-analytical processes, especially human error. It was found that among 207 PND requests for Hb Bart’s hydrops fetalis, 84 (40.6%) had one or both parents with heterozygous Hb E whose Hb E ≥ 25%, and 24 (11.6%) PND requests of one or both parents with MCV ≥ 80 fL. The cut-off values for Hb E and MCV have been used effectively to rule out α^0^-thalassemia in an ongoing screening strategy in the region [6, 7, 42]. PND of Hb Bart’s hydrops fetalis in these cases is therefore unnecessary. Therefore, it is not unexpected that among these 294 PND requests, no affected fetus was identified. In addition, further consideration of the overall PND outcomes of these 645 fetuses in this unnecessary PND group identified 114 (17.7%) affected, 260 (40.3%) unaffected carriers, and 271 (42.0%) normal fetuses. In fact, it is possible that number of PND requests with no risk of thalassemia disease might be more than 294 as presented in the unnecessary PND group. There were as many as 409 PND requests with inadequate data for fetal risk assessment. The outcomes of these 409 PND in this group revealed 53 (13.0%) affected fetuses, 145 (35.5%) unaffected carriers, and 211 (51.6%) normal fetuses, much different from the expected theoretical values for a recessive genetic disorder. This indicates likely that many fetuses should have no risk for thalassemic diseases.

Nonetheless, our results indicate that unnecessary PND is not uncommon in routine practice. Apart from the above-mentioned causes, other etiologies may include delayed antenatal care with advanced gestational age, inadequate fetal risk assessment by Hb analysis without DNA analysis of the parents, requests without paternal specimens, laboratory errors, and lack of knowledge in laboratory interpretation. It is essential to understand this unwanted event in PND of hemoglobinopathies in order to reduce the unnecessary complications related to fetal specimen collection, psychological impacts on pregnant women and their families, as well as expense and workload of PND.

## Supporting information

S1 Table α-thalassemia mutations (a total of 3,104 alleles) identified among 1,520 couples at risk of having fetuses with Hb Bart’s hydrops fetalis and 32 couples at-risk for Hb H disease. (DOC)

S2 Table β-thalassemia mutations identified among 2,555 at-risk couples for β-thalassemia diseases including β^0^- thalassemia (2,479 alleles) and β^+^-thalassemia (224 alleles) and unknown (24 alleles). (DOC)

S3 Table Molecular basis of high Hb F determinants found among 115 at-risk couples with a total of 122 alleles. (DOC)

S4 Table Abnormal hemoglobins encountered among 16 at-risk couples. (DOC)

S1 Raw data Raw data used in Tables 2 and 3, and S1–S4 Tables. (XLSX)
