# Supplementary material for: Frequency of unnecessary prenatal diagnosis of hemoglobinopathies: A large retrospective analysis and implication to improvement of the control program
Source: PLoS One. 2023 Apr 14;18(4):e0283051. doi: 10.1371/journal.pone.0283051 (PMC10104333; doi:10.1371/journal.pone.0283051)
Supplement: S1 Table — (DOC) [file pone.0283051.s001.doc]

**S1 Table.** α-Thalassemia mutations (a total of 3,104 alleles) identified among 1,520 couples at risk of having fetuses with Hb Bart’s hydrops fetalis and 32 couples at-risk for Hb H disease.

| **α-thalassemia mutations** | **HGVS name** | **Number of alleles (%)** |
| --- | --- | --- |
| **α^0^-thalassemia** |  | **3,072 (99.0)** |
| α^0^-thalassemia (SEA deletion) | NC_000016.10:g.165401_184701del | 3,063 (98.7) |
| α^0^-thalassemia (THAI deletion) | NC_000016.10:g.149863_183312del | 8 (0.3) |
| Unknown α^0^-thalassemia | - | 1 (0.0) |
| **α^+^-thalassemia** |  | **32 (1.0)** |
| α^+^-thalassemia (3.7 kb deletion) | NG_000006.1:g.34247_38050del | 16 (0.5) |
| Hb Constant Spring | HBA2:c.427T>C | 14 (0.5) |
| Hb Paksé | HBA2:c.429A>T | 1 (0.0) |
| Hb Quong-Sze | HBA2:c.377T>C | 1 (0.0) |
| **Total** |  | **3,104** |
