# Supplementary material for: Frequency of unnecessary prenatal diagnosis of hemoglobinopathies: A large retrospective analysis and implication to improvement of the control program
Source: PLoS One. 2023 Apr 14;18(4):e0283051. doi: 10.1371/journal.pone.0283051 (PMC10104333; doi:10.1371/journal.pone.0283051)
Supplement: S2 Table — (DOC) [file pone.0283051.s002.doc]

**S2 Table.** β-Thalassemia mutations identified among 2,555 at-risk couples for β-thalassemia diseases including β^0^- thalassemia (2,479 alleles) and β^+^-thalassemia (224 alleles) and unknown (24 alleles).

| **β-thalassemia mutations** | **HGVS name** | **Number of alleles (%)** |
| --- | --- | --- |
| **β^0^-thalassemia** |  | **2,479 (90.9)** |
| CD41/42 (-CTTT) | HBB:c.126_129delCTTT | 1053 (38.6) |
| CD17 (A>T) | HBB:c.52A>T | 715 (26.2) |
| IVSI-1 (G>T) | HBB:c.92+1G>T | 195 (7.2) |
| IVSI-5 (G>C) | HBB:c.92+5G>C | 115 (4.2) |
| 3.4 kb del | NC_000011.10:g.5224302_5227791del3490bp | 111 (4.1) |
| CD71/72 (+A) | HBB:c.217dupA | 100 (3.7) |
| IVSII-654 (C>T) | HBB:c.316-197C>T | 71 (2.6) |
| CD35 (C>A) | HBB:c.108C>A | 30 (1.1) |
| CD41 (-C) | HBB:c.126delC | 15 (0.6) |
| CD27/28 (+C) | HBB:c.85dupC | 13 (0.5) |
| CD26 (G>T) | HBB:c.79G>T | 12 (0.4) |
| 105bp del | HBB:c.-74_31del | 10 (0.4) |
| Filipino deleletion | NC_000011.10:g.5112882 _5231358del | 8 (0.3) |
| CD15 (-T) | HBB:c.46delT | 8 (0.3) |
| CD95 (+A) | HBB:c.287dupA | 5 (0.2) |
| CD 123-125 (-ACCCCACC), Hb Khon Kaen | HBB:c.370_378delACCCCACCA | 5 (0.2) |
| CD43 (G>T) | HBB:c.130G>T | 4 (0.1) |
| Init CD ATG>AGG | HBB:c.2T>G | 3 (0.1) |
| IVS1-116 (T>G) | HBB:c.93-15T>G | 2 (0.1) |
| CD30 (G>C) | HBB:c.93G>C | 1 (0.0) |
| CD33/34 (-G) | HBB:c.102_103delG | 1 (0.0) |
| CD121 (-G) | HBB:c.364delG | 1 (0.0) |
| IVSII-1 (G>A) | HBB:c.315+1G>A | 1 (0.0) |
| **β^+^-thalassemia** |  | **224 (8.2)** |
| NT-28 (A>G) | HBB:c.-78A>G | 110 (4.0) |
| CD19 (A>G), Hb Malay | HBB:c.59A>G | 77 (2.8) |
| NT-31 (A>G) | HBB:c.-81A>G | 26 (1.0) |
| CD126 (T>G), Hb Dhonburi | HBB:c.380T>G | 7 (0.3) |
| NT-87 (C>A) | HBB:c.-137C>A | 2 (0.1) |
| NT-30 (T>C) | HBB:c.-80T>C | 1 (0.0) |
| NT-86 (C>G) | HBB:c.-136C>G | 1 (0.0) |
| **Unknown β-thalassemia** |  | **24 (0.9)** |
| **Total** |  | **2,727** |
