# Supplementary material for: Frequency of unnecessary prenatal diagnosis of hemoglobinopathies: A large retrospective analysis and implication to improvement of the control program
Source: PLoS One. 2023 Apr 14;18(4):e0283051. doi: 10.1371/journal.pone.0283051 (PMC10104333; doi:10.1371/journal.pone.0283051)
Supplement: S3 Table — (DOC) [file pone.0283051.s003.doc]

**S3 Table.** Molecular basis of high Hb F determinants found among 115 at-risk couples with a total of 122 alleles.

| **High Hb F determinants** | **HGVS name** | **Number of alleles (%)** |
| --- | --- | --- |
| **- δβ^0^-thalassemia** (12.6 kb deletion) | NG_000007.3:g.64384_76993del | 98 (80.3) |
| **- HPFH-6** | NC_000011.10:g.5172745_5252029del | 18 (14.8) |
| **- Indian deletion-inversion ^A^γδβ^0^-thalassemia** | NG_000007.3:g.48400_49245del;49246_64567inv; 64568_72051del | 2 (1.6) |
| **- Thai deletion-inversion-insertion ^A^γδβ^0^-thalassemia** | NG_000007.3:g .47449_165744del;168412_168590invins | 2 (1.6) |
| **- SEA-HPFH** | NC_000011.10:g.5201647_5229059del | 2 (1.6) |
| **Total** |  | **122** |
