# Supplementary material for: Frequency of unnecessary prenatal diagnosis of hemoglobinopathies: A large retrospective analysis and implication to improvement of the control program
Source: PLoS One. 2023 Apr 14;18(4):e0283051. doi: 10.1371/journal.pone.0283051 (PMC10104333; doi:10.1371/journal.pone.0283051)
Supplement: S4 Table — (DOC) [file pone.0283051.s004.doc]

**S4 Table.** Abnormal hemoglobins encountered among 16 at-risk couples.

| **Abnormal Hb** | **HGVS name** | **Number of alleles (%)** |
| --- | --- | --- |
| Hb Tak | HBB:c.440_441dupAC | 4 (25.0) |
| Hb Lepore | NG_000007.3:g.63290_70702del | 4 (25.0) |
| Hb Hope | HBB:c.410G>A | 2 (12.5) |
| Hb Pyrgos | HBB:c.251G>A | 2 (12.5) |
| Hb J-Bangkok | HBB:c.170G>A | 2 (12.5) |
| Hb C | HBB:c.19G>A | 1 (6.3) |
| Hb Korle-Bu | HBB:c.220G>A | 1 (6.3) |
| **Total** |  | **16** |
